# Improving Confidence and Self-Esteem Among Socioeconomically Disadvantaged Children: A Social Emotional Learning Intervention in Rural China

**DOI:** 10.3390/bs15101352

**Published:** 2025-10-02

**Authors:** Jiameng Li, Lin Zhu, Therese Hesketh

**Affiliations:** 1 School of Psychology, Shanghai Normal University, 100 Guilin Road, Xuhui District, Shanghai 200234, China; jiamengli@zju.edu.cn; 2 School of Tourism, Shanghai Normal University, 100 Guilin Road, Xuhui District, Shanghai 200234, China; 3 Shanghai Health Development Research Center (Shanghai Medical Information Center), 1477 Beijing West Road, Jingan District, Shanghai 200031, China; 4 Key Discipline of Zhejiang Province in Public Health and Preventive Medicine (First Class, Category A), Hangzhou Medical College, Yikang Street, Linan District, Hangzhou 311300, China; 5 School of Medicine, Southern University of Science and Technology, 1088 Xueyuan Avenue, Shenzhen 518055, China

**Keywords:** social emotional learning (SEL), self-esteem, confidence, economically disadvantaged Chinese children, mixed-methods

## Abstract

Background: Children in underdeveloped rural areas of China often face socioeconomic disadvantages, which are associated with low confidence and self-esteem. While SEL programs have shown benefits internationally, evidence from Mainland China is limited. This study examined whether a school-based SEL intervention could improve confidence and self-esteem among children in economically disadvantaged rural areas. Methods: The intervention was a quasi-experimental study conducted in a rural, underdeveloped region of central China. It involved 16 weekly sessions, each lasting 90 min. A total of 230 children aged 8–12 years participated in the intervention school, while 325 children from another school served as the control group. The study used a mixed-methods design, including a quantitative survey administered at baseline, post-intervention, and a 5-month follow-up, as well as qualitative interviews with 83 children, nine caregivers, and eight teachers following the intervention. A linear mixed-effects model was employed to evaluate the effectiveness of the intervention, while interview data were analyzed using an inductive thematic approach. Results: The findings suggested (1) children from lower socioeconomic backgrounds consistently reported lower levels of self-esteem and self-efficacy across all three assessment points. (2) There was a short-term intervention effect on self-esteem, with greater improvements among children from poorer families. (3) The increase in children’s confidence meant they were more able to express themselves and mix with others. (4) Children’s improvements were not sustained up to a 5-month follow-up. Conclusions: The program may be effective in improving children’s confidence and self-esteem in underdeveloped rural areas of China. Such a program may contribute not only to educational outcomes but also to broader efforts aimed at social mobility and poverty reduction.

## 1. Introduction

Self-esteem is typically defined as an individual’s evaluation of their overall worth as a person (Steiger et al., 2014). Domain-specific self-esteem refers to an individual’s self-evaluation in a specific domain, such as intellectual ability, physical appearance, and social competence. Self-confidence is defined as an individual’s trust in his or her abilities, capacities, and judgments, or the belief that he or she can successfully face day-to-day challenges and demands (Ackerman, 2023). Higher levels of confidence and self-esteem are associated with better well-being and contribute to achieving personal goals (Dinos & Palmer, 2015; Silverthorn et al., 2017; Wilkinson & Kao, 2019). In addition, higher socio-economic status has been shown to be correlated with confidence and self-esteem (Twenge & Campbell, 2002). A large body of research in children and adolescents has shown associations between low self-esteem and a range of negative outcomes, including academic difficulty, internalizing mental disorders (e.g., depression, anxiety, eating disorders, and suicide attempts) (Sowislo & Orth, 2013; Steiger et al., 2014), externalizing mental disorders (e.g., violence, aggressive behaviors), and risk behaviors (e.g., alcohol and tobacco use) (Arsandaux et al., 2021; Trzesniewski et al., 2006).

### 1.1. SEL-Based Interventions to Enhance Confidence and Self-Esteem

There has been increasing interest in interventions aimed at strengthening confidence and self-esteem among children and adolescents (Silverthorn et al., 2017). Social emotional learning (SEL) is the process through which social emotional competence develops (Domitrovich et al., 2017). SEL promotes five interrelated cognitive, affective, and behavioral competencies, which are important for success in school and life: (1) self-awareness (e.g., recognizing feelings, strengths and weaknesses), (2) self-management (e.g., managing emotions and behaviors), (3) social awareness (e.g., empathy and understanding the perspective of others), (4) relationship skills (e.g., developing and keeping positive relationships), and (5) responsible decision making (Weissberg et al., 2015). SEL is thus a comprehensive program that covers a wide range of topics and social competencies, which can provide children with a holistic approach to personal growth and development. A school-based SEL intervention has been shown to enhance children’s self-awareness, which includes confidence and self-esteem as key components (Fu et al., 2024). Meta-analyses have shown that SEL interventions can enhance children’s self-esteem and confidence across diverse settings (Durlak et al., 2011). Evidence also suggests these benefits may be particularly pronounced among children from disadvantaged backgrounds, who often begin with lower baseline levels of self-esteem (Veríssimo et al., 2022). However, very few studies in Mainland China have examined these specific outcomes, creating a critical research gap.

Many SEL programs are informed by cognitive-behavioral principles (de Jonge-Heesen et al., 2020), which provide specific techniques to help children reshape negative self-beliefs and strengthen their self-esteem.

### 1.2. Cognitive Behavioral Approach in SEL-Based Interventions

The cognitive behavioral approach has been referred to as the theoretical basis for some SEL-based interventions (Barrett & Turner, 2001; de Jonge-Heesen et al., 2020; Lowry-Webster et al., 2003). Guided by this approach, participants are taught to identify how activating events, thoughts, emotions, and behaviors are related (de Jonge-Heesen et al., 2020; Miller et al., 2017). The cognitive behavioral approach represents a technique in which psychological distress and maladaptive behavior are treated by changing people’s thinking style and behavior (Hyun et al., 2005). The group format cognitive behavioral approach is now widely used for children and adolescents, and it helps them identify and face personal challenges (Epel et al., 2021). Low self-esteem is associated with children’s negative perceptions about themselves, and the cognitive behavioral approach has been shown to be effective in increasing self-esteem in some individuals (Taylor & Montgomery, 2007).

In this study, the cognitive-behavioral approach provides the theoretical framework for understanding how SEL may enhance confidence and self-esteem. Low self-esteem is often associated with negative self-perception and maladaptive thought patterns (Taylor & Montgomery, 2007). By teaching children to identify and reframe such thoughts, CBT-based strategies within SEL curricula provide mechanisms for improving self-perception, building confidence, and promoting adaptive behavior. This framework underpinned the intervention design and guided our focus on confidence and self-esteem as key outcomes.

### 1.3. SEL in Mainland China

There is strong evidence of the effectiveness of school-based SEL programs in improving children’s well-being from a number of countries (Kuosmanen et al., 2019; Sklad et al., 2012). However, evidence from Mainland China on the feasibility and effectiveness of SEL programs is limited. In 2011, the Ministry of Education of China, in collaboration with UNICEF, initiated a pilot SEL program in five Chinese provinces (Guizhou, Yunnan, Chongqing, Guangxi, and Xinjiang), with the aim of improving children’s self-awareness, emotion management, motivation, empathy, and social skills (UNICEF, 2020). However, information about the program’s implementation and results has not been reported. In recent years, there has been a growing interest in implementing and enhancing the effectiveness of SEL programs in Mainland China (Fu et al., 2024; Li & Hesketh, 2023).

### 1.4. The Strong Emphasis on Academic Achievement in China

China’s education system is characterized by intense competition and a strong societal focus on academic performance, shaping children’s self-perception from an early age. Most parents in China attach great importance to children’s academic achievement, which is seen as the most effective way to achieve upward social mobility (Chui & Wong, 2017). This contributes to a highly competitive and stressful educational experience. Chinese primary school children spend about eight hours in school and four hours doing homework from Monday to Friday, with additional homework on the weekends (Stewart & Sun, 2007). In underdeveloped regions of rural China, academic achievement is widely regarded as a primary way to escape poverty, and thus, admission rates to good high schools have become the only focus of many rural schools (Pan & Ye, 2017). This means that extracurricular activities of all types are often neglected, and rural children are in an even worse situation than their urban peers in terms of the development of social-emotional skills.

### 1.5. The Necessity and Main Aims of the Current Study

Although China has made significant progress in poverty reduction over the past decades, many rural communities experience social and economic decline due to rapid urbanization. Raising the most disadvantaged families out of poverty remains a significant challenge in rural China. Children living in underdeveloped rural regions of China often face socioeconomic disadvantages, including limited access to educational and psychological resources, inadequate parental support due to labor migration, and a lack of extracurricular opportunities (Yu et al., 2025). These factors can contribute to emotional and developmental challenges, particularly low self-confidence and poor self-esteem. Previous research has shown that children living in poverty are at higher risk of experiencing negative self-perceptions, which in turn can impact their academic engagement, social functioning, and overall well-being (Doi et al., 2019). Given this context, there is a critical need for culturally responsive, school-based interventions that address the social-emotional needs of these children. Interventions such as SEL could play a critical role in improving children’s long-term life chances and educational trajectories. This study was therefore conducted in economically underdeveloped rural areas to examine the effectiveness of a localized SEL program designed to enhance children’s confidence and self-esteem.

Previous studies of SEL programs predominantly used quantitative methods to measure the intervention effects, and very few carried out qualitative interviews (Clarke et al., 2015; Skryabina et al., 2016). For this study, both quantitative and qualitative methods were used to gain an in-depth understanding of whether an SEL program can benefit children in underdeveloped rural China. Although SEL programs have been associated with a range of outcomes, including social skills, emotional awareness, and emotion regulation, this paper specifically examines how an SEL program can influence confidence and self-esteem. These constructs are central to children’s self-perception and motivation, shaping how they engage with academic and social challenges. For children growing up in rural poverty, low confidence and self-esteem may restrict classroom participation, limit peer interactions, and reinforce feelings of inferiority compared to more advantaged peers (Doi et al., 2019). Conversely, building confidence and self-esteem can empower children to express themselves, persist in learning, and envision broader possibilities for their future. By focusing on these outcomes, the present study aimed to explore whether (1) there is an association between family economic status and children’s self-esteem and confidence, (2) a school-based SEL program could improve children’s self-esteem and confidence, and (3) there is behavior change in children following the SEL program based on their narratives.

## 2. Materials and Methods

The research employed a mixed-methods approach with a control group in a quasi-experimental design.

### 2.1. Intervention Implementation

The intervention took place in two primary schools located in underdeveloped villages in central China (Nanyang, Henan province). It was delivered across 16 weekly sessions, incorporated into the regular school timetable, from September 2021 to January 2022. Each session comprised two 45 min lessons, with a 10 min break between them. We recruited six graduate students from the psychology department of a local university to serve as facilitators. Each facilitator was assigned to one class and led the same group of approximately 50 children throughout the program, corresponding to the typical class size in the intervention school. Facilitators received training in basic SEL knowledge, program goals, the content, and organization of activities. Facilitators were provided with ongoing support and feedback after each session from the program coordinators, which was important to the quality of the program implementation.

Four of the 16 sessions focused on enhancing confidence and self-esteem, covering topics such as recognizing personal strengths, expressing oneself effectively, setting and achieving personal goals, and developing a positive self-image. During the program, children were instructed in a range of cognitive behavioral skills that underpin the framework of social emotional learning. These included identifying negative thinking, finding positive alternatives, increasing confidence, regulating emotions, communicating effectively, perspective taking, and developing healthy interpersonal relationships. The sessions incorporated various child-focused activities such as group discussions, role-playing, positive self-talk, drawing, storytelling, video viewing, collaborative crafts, and educational games. The program was primarily adapted from the Chinese Ministry of Education–UNICEF’s social-emotional learning program (UNICEF, 2020). One example of the adaptation process is that many children in this study lacked confidence, both in the classroom and in social situations. To deal with this, videos of popular cartoons, focusing on confidence, were shown, and then the facilitators led a discussion on the cartoons. In the SEL classes, children were taught skills to identify thinking patterns that reduce confidence and to replace them with positive thoughts that increase confidence. Children were also taught to develop positive self-talk, including praising or complimenting oneself. In addition, reflecting on their own positive attributes helps children develop a stronger sense of self-esteem. Therefore, each child was given a small stack of slips of paper and pens. Children were asked to write or draw something they like about themselves, with prompts like, “What’s something you’re proud of?” or “What’s a talent you have?”

A total of 145 activities were carried out as part of the program. Implementation fidelity was assessed using facilitators’ checklists, which recorded the number and percentage of activities that were fully, partially, or not implemented (Li & Hesketh, 2023). Across all six grades, the overall full implementation rate exceeded 90%, with third grade (97.2%) and sixth grade (96.6%) achieving the highest fidelity. These results indicate a high level of implementation fidelity, which strengthens the internal validity of the study. SEL classes were integrated into the intervention school’s curriculum and implemented during self-study sessions every Wednesday afternoon. As the program was part of the regular school schedule, student attendance remained consistently high, exceeding 95% in each session.

### 2.2. Participants

The program was conducted in two underdeveloped villages in Henan province in central China. Initially, the local education bureau recommended five schools in villages, which were the lowest in the ranking of household annual income of the area. All five schools were willing to participate, and two were randomly selected. After obtaining permission from the schools, participants were randomly assigned to either the intervention or the wait-list control. The two schools shared similar characteristics; for example, both had an average class size of around 50 children. Both schools face significant challenges in school teacher recruitment, with most teachers employed on short-term contracts and recruited from nearby towns or cities. Due to the persistent teacher shortage, instruction is limited to core subjects such as Chinese, mathematics, and English, while subjects like physical education, music, and art, though officially part of the national curriculum, are not offered. Additionally, approximately 40% of the students are “left-behind children”, whose parents have migrated to urban areas in search of work to escape poverty, leaving the children primarily in the care of grandparents. School records indicated that students in both schools came from low-income households. In 2021, approximately 80% had a per capita annual household income of 10,000 RMB (about $1393) or less, a figure significantly below the rural national average of 18,931 RMB (around $2637) (National Bureau of Statistics, 2022). The two schools are located 5 km from each other, with different catchment areas, and no overlap in the villages they cover, so children had little contact with those from different villages, preventing potential contamination between the schools.

All students from the six grades in the intervention school took part in the program. However, first-grade students aged 7 were excluded from the questionnaire-based surveys due to difficulties in comprehending the questions. A total of 555 students were included in the quantitative sample, with 230 (46% male) from the intervention school and 325 (51% male) from the control school. The response rate for completing all three assessments was 97.4% (555 out of 570). Qualitative interviews were conducted in the intervention school with three groups of participants: school teachers, caregivers, and children.

### 2.3. Data Collection and Measurements

Surveys, using the same self-administered questionnaire for all participating children, were conducted at three time points: one week before the intervention (pre-test, Assessment 1), one week after the intervention ended (post-test, Assessment 2), and five months following the completion of the intervention (follow-up, Assessment 3). The final questionnaire had three parts: (1) Sociodemographic and background details: sex, age, grade level, whether living with parents, parents’ occupations, parents’ migration status, family economic status, and academic performance. (2) The Rosenberg Self-Esteem Scale (RSES), the most commonly used measure of self-esteem in psychology research, was used to assess children’s self-esteem (Rosenberg, 1965). The RSES consists of 10 items, and four of them were included in this study. The selection of these four items was informed by feedback from children in the pilot, indicating that these four were straightforward and directly related to their personal experience, and thus, more effective in eliciting reliable responses from the children. Each item is scored 1 to 4, representing strongly disagree to strongly agree, with a higher score indicating better self-esteem (Jiang et al., 2023). Internal consistency of the self-esteem scale was acceptable at all three time points (Cronbach’s α = 0.75 at baseline, 0.79 at post-test, and 0.83 at follow-up). To examine whether the scale functioned equivalently over time, longitudinal measurement invariance was tested using confirmatory factor analysis. The configural model demonstrated acceptable fit (CFI = 0.952, RMSEA = 0.064, SRMR = 0.041), indicating that the factor structure was consistent across the three waves. Constraining factor loadings equal across time (metric invariance) did not reduce model fit (ΔCFI = 0, ΔRMSEA = 0, ΔSRMR = 0), supporting metric invariance. Further constraining item intercepts to equality (scalar invariance) also had a negligible impact on fit (ΔCFI = 0, ΔRMSEA = 0, ΔSRMR = −0.003), indicating scalar invariance. These results suggest that the scale can be used to compare latent means across the three time points. (3) Although confidence and self-efficacy are not identical concepts, self-efficacy is widely regarded as a relevant proxy for confidence, particularly when domain-specific confidence measures are unavailable. Given this, the general self-efficacy scale (GSES) was used in this study to assess children’s confidence levels. The GSES consists of 10 items (three of them were included in this study) that evaluate how confident individuals feel about their ability to manage tasks and setbacks in everyday life (Schwarzer & Jerusalem, 1995). Respondents rate their agreement with each statement on a 4-point scale, ranging from “Not at all true” to “Exactly true”. Higher scores reflect a stronger belief in personal efficacy. Cronbach’s alpha was 0.64 at baseline, 0.58 at post-test, and 0.68 at follow-up. The Cronbach’s α values in our study were somewhat lower than those typically reported for the full 10-item GSES. This is likely due to a shortened version of the GSES being used, and Cronbach’s α is known to decrease as the number of items decreases (Romppel et al., 2013). To assess whether the scale operated equivalently over time, longitudinal measurement invariance was tested using confirmatory factor analysis. The configural model demonstrated good fit (CFI = 0.982, RMSEA = 0.034, SRMR = 0.029), indicating that the factor structure was consistent across the three waves. Constraining factor loadings equal across time (metric invariance) did not reduce model fit (ΔCFI = 0, ΔRMSEA = 0, ΔSRMR = 0), supporting metric invariance. Further constraining item intercepts (scalar invariance) also resulted in negligible changes in model fit (ΔCFI = 0, ΔRMSEA = 0, ΔSRMR = −0.002), indicating scalar invariance. These results suggest that the scale can be used to compare latent means across the three time points.

Self-esteem is generally regarded as a more stable trait, whereas confidence tends to fluctuate depending on specific contexts and tasks. While we measured general self-efficacy quantitatively, a qualitative approach was also used to explore confidence more deeply, as its situational nature makes it challenging to capture comprehensively using standardized scales. Semi-structured interviews were conducted face-to-face during the follow-up assessment to explore the thoughts of children, caregivers, and school teachers regarding the program. School teachers were asked about (1) changes in the overall confidence or self-esteem of children following the SEL classes and (2) how long any of the changes lasted. Caregivers were asked about any changes in their child’s overall confidence or self-esteem following the SEL classes, as well as whether the program had benefited their child. Children were asked about (1) changes in their overall confidence or self-esteem following the SEL classes, (2) what activities and factors related to the SEL program influenced them. Interviews were carried out by the program coordinator, who was not directly engaged in the delivery of the program. Interviews were held one-to-one in a private room at the school. The duration of the interviews ranged from 30 to 45 min. Interviews were recorded in audio format and transcribed exactly as spoken, with the participants providing written consent. The qualitative interviews were originally conducted in Chinese and subsequently translated into English for analysis and interpretation. To ensure the accuracy and fidelity of the translations, a back-translation process was employed. The back-translated Chinese versions were compared with the original transcripts to identify and resolve any discrepancies.

### 2.4. Ethics

Ethical approval was obtained from the Zhejiang University Ethics Committee before conducting the program. Written informed consent was obtained from all the participants, including children and their caregivers. Both paper questionnaires and interview audio recordings were anonymously coded according to participants’ ID numbers and stored confidentially.

### 2.5. Data Analyses

First, baseline differences in background information between the intervention and control groups were assessed using chi-square tests and *t*-tests. Independent-samples *t*-tests were conducted to examine differences in self-esteem and self-efficacy between groups (intervention vs. control, and across levels of family economic status) at the three assessments. Second, linear mixed-effects regression modeling (LMM) was employed to evaluate the impact of the intervention on self-esteem and self-efficacy, with repeated measures nested within subjects. We specified random intercepts for subjects and fixed effects of condition (intervention vs. control) and time (assessments). Third, to assess family economic status as a potential moderator of the intervention effect on self-esteem, the interaction between condition (intervention vs. control) and family economic status (above average vs. average or below) was examined using a linear model, with changes in self-esteem scores as the dependent variable. Data analyses were performed with R 4.2.1.

We conducted an inductive thematic analysis (Braun & Clarke, 2006). After carefully reviewing the interview transcripts, we proceeded through several stages: (1) generating initial codes, e.g., volunteer to answer teachers’ questions, express oneself, more able to mix with others, more friends, (2) organizing these codes into candidate themes, (3) compiling all relevant extracts under each theme, and (4) refining and reviewing the themes. This procedure was applied consistently across data from children, teachers, and caregivers (Clarke et al., 2015).

## 3. Results

### 3.1. Sociodemographic and Background Information

Table 1 reports the baseline sociodemographic and background characteristics by group, along with chi-square tests of group differences. At baseline, we found few differences between the groups. However, fewer children in the intervention group lived with both parents (*p* = 0.01), with more children in the intervention group having a migrant father (*p* = 0.02). The variable “father/mother migration” in this report also includes those whose parents migrate on an intermittent basis, for short periods of time, for example, living with their children during the baseline survey but migrating for work for a few months following this.

### 3.2. The Scores of Self-Esteem and Self-Efficacy at Three Assessments

Table 2 presents the descriptive statistics for self-esteem and self-efficacy scores by treatment group (control vs. intervention) and family economic status (above average vs. average or below) at each assessment. Independent-samples *t*-tests were conducted to examine differences in self-esteem and self-efficacy scores between groups. At baseline, there was no significant difference between the intervention and control groups for self-esteem and self-efficacy. Children from lower family economic status reported significantly lower self-esteem and self-efficacy scores across all three assessments compared to their peers from higher economic status.

### 3.3. Intervention Effects on Self-Esteem and Self-Efficacy

The effects of intervention on self-esteem and self-efficacy are presented in Table 3. They are conducted by three linear mixed models (LMMs), adjusting for sex, grade, and whether living with parents.

A linear mixed model (LMM) showed a significant condition × time interaction effect of self-esteem at post-intervention. Compared to changes in self-esteem scores from Assessment 2 to 1 in the control group, the intervention group reported higher scores at Assessment 2, B = 0.57, 95% CI [0.07, 1.07], *p* = 0.025, than at Assessment 1. While there was no significant interaction effect of self-esteem at Assessment 3. It suggested that the intervention school had improved self-esteem in children compared to the control school from baseline to post-intervention, but this improvement did not persist to the 5-month follow-up.

There was no significant condition × time interaction effect of self-efficacy. There was no difference between intervention and control groups in the changes in self-efficacy scores from Assessment 2 to 1, B = −0.32, 95% CI [−0.77, 0.13], *p* = 0.16, or from Assessment 3 to 1, B = −0.28, 95% CI [−0.73, 0.17], *p* = 0.47.

Given that the intervention had an effect on self-esteem but not on self-efficacy, the change in self-esteem scores from Assessment 2 to 1 was selected as the dependent variable to examine the moderating effect of family economic status. The model showed “Condition × Family economic status” interaction short-term effect on self-esteem, B = 1.58, 95% CI [0.46, 2.69], t = 2.77, *p* = 0.006, which implied that compared to children from richer families, those from poorer families in the intervention school reported higher self-esteem at post-intervention relative to baseline, as compared to the control school.

In summary, the intervention had a short-term effect on self-esteem, with stronger improvements observed among children from lower-income families, indicating that family economic status moderated the intervention effect. No significant intervention effect was found for self-efficacy.

### 3.4. Qualitative Interviews

All eight school teachers from 2nd to 6th grade in the intervention school participated in interviews. The average age of these teachers was 38 years (from 26 to 52). One had received a high school education, and seven had received a college education. Nine caregivers, including five mothers and four grandmothers, volunteered to be interviewed. The average age of the caregivers was 44 years (from 35 to 57). Six caregivers had received a secondary school education, and three had received a primary school education. Six were homemakers, and three worked part-time. We planned to randomly select 30% of children from the list in each class, a sample size expected to be sufficient to reach data saturation. In the end, we interviewed 83 children (36 boys and 47 girls) in the intervention school. The interviewed children were aged 8–12 years, and 40% were left-behind children (children of migrant workers), mainly looked after by grandparents.

**Figure 1:**
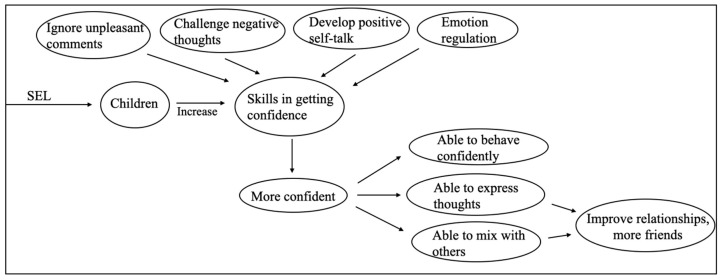
Conceptual framework of the SEL intervention on the change in children’s confidence.

More than one-third (n = 34) of the interviewed children reported they felt more confident following the SEL classes. Seven of the eight interviewed school teachers and six of the nine interviewed caregivers reported improvements in children’s confidence since the SEL classes. Two main themes emerged from the interviews: (1) Changes in children’s confidence and self-esteem. (2) Elements of the program that improved children’s confidence. The results of the interviews provided insights for a conceptual framework, which is illustrated in Figure 1.

Theme 1: Changes in children’s confidence

Children were able to behave confidently, express their thoughts and feelings, and interact more effectively with others following the SEL classes. Several children reported feeling generally better about themselves. A 12-year-old boy reported, “After the SEL classes, I became confident and happy.”

Behaving more confidently

Five children reported they were able to act more confidently. A girl, aged 10, described this well: “*In the past, if there was an activity in my class, I didn’t put myself forward, but now I usually volunteer.*” An 8-year-old girl stated, “*Before I wasn’t confident at all. I didn’t say anything in class, but now I raise my hand to answer questions.*”

Three teachers stated that children were more comfortable answering questions in their classes. A 4^th^-grade teacher said, “Some children in my class are much more confident since the SEL classes. In the past, most of them were very introverted, but now they speak out and more of them answer my questions. This motivates me to improve my teaching.” Two teachers revealed that although the SEL classes brought positive change, the effects did not last long after the end of the program. A 5th-grade Chinese teacher said, “Overall, one third of the children in my class showed clear benefits, for example, some children had never answered my questions and didn’t chat with other children, but they do now. One girl really surprised me. She was very introverted and never answered questions before, but after the SEL classes, she even did presentations in front of the whole class. But two months after the end of the SEL classes, she is quiet again. If the SEL classes could continue, we might see long-term effects.”

Two caregivers mentioned that their children exhibited greater confidence after attending the SEL classes. A mother of three children observed, “*My younger son is more confident now. In the past, he thought he couldn’t do anything well and never as well as his two elder sisters, but the SEL classes changed that. At least, he tries now.*”

Expressing thoughts and feelings

Three children reported that since the SEL classes, they were more able to say “no” when they felt uncomfortable. Five children revealed that the SEL classes helped them to express themselves confidently. A 11-year-old girl said, “*In the past, I always kept things to myself, but now I’m able to speak out confidently.*”

Five teachers mentioned that children were more able to express their thoughts and feelings. A 2nd-grade teacher revealed, “*The SEL classes helped children to communicate with each other.*” A 3rd-grade teacher reported, “*Children have become more outgoing, and don’t just keep things to themselves.*” A 5th-grade math teacher stated, “*Children were encouraged to express themselves in the SEL classes. So now they are more able to communicate their feelings and thoughts.*”

Three caregivers also noticed that children were more able to express their thoughts. A mother of two daughters said, “My younger daughter used to think that she wasn’t as good as her sister and was afraid of saying what she thought, but now she’s much better. I know the SEL teacher always encourages her to express her feelings, so I have learnt to encourage her, too. And she doesn’t feel inferior to her sister anymore.” The mother of a boy aged 9 reported, “My son is much more confident now. When I ask about his scores in tests, he always says he is doing well, but before, he said he wasn’t.”

Mixing with others

Six children reported that they were much more comfortable talking and playing with others. A boy aged 12 stated, “*In the past, I never played with others during the class break, but now I do. I have made friends.*” A girl aged 10 said, “*I was quite shy before, and found it difficult to go up to classmates and chat. I’m much better now, and I really like mixing with others.*”

The observation that children were more able to mix with others was made by three caregivers. The mother of a girl aged 12 stated, “*My daughter changed a lot in personality following the SEL classes. She became outgoing and confident. Before she hardly talked to anyone or mixed with others, and didn’t have friends, but now she talks more and has made a few friends, who even come to our home to play.*”

Theme 2: Elements of the program that improved children’s confidence

The children thought that role-playing and discussion of stories helped them to think in more positive ways, which in turn increased self-esteem and confidence. The classes on managing emotions also helped them deal with nervousness and anxiety, which helped them to overcome low levels of confidence.

Ignoring unpleasant comments

Three children described feeling more able to ignore unpleasant comments from others. A 11-year-old boy reported, “*I don’t care what others say now, when someone makes* horrible *comments about me, I just ignore them.*” A girl, aged 9, said, “*My desk mate used to criticize my painting, so I lost confidence in my painting and didn’t enjoy it. After the SEL classes, I just ignore him and now I feel good about my painting.*”

Challenging negative thoughts and beliefs

Five children reported that since taking the SEL classes, they were aware of being able to actively stop negative thoughts and focus on positive alternatives. A girl aged 9 revealed, “*I thought I was stupid and blamed myself if I didn’t do well in exams. But in the SEL classes I found it doesn’t matter even if I don’t get good scores in exams. It is just important to try hard and I don’t worry any more.*”

Developing positive self-talk

Three children reported that complimenting themselves, as is taught in the SEL classes, made them more confident. A girl aged 12 stated, “*Every day I praise myself for something, and tell myself I’m very good, which increases my confidence.*” Two children reported that positive self-talk made them feel motivated, especially when something did not go well. A boy aged 11 reported, “*Now if I fail a test, I tell myself I can do better next time.*”

Regulating emotions

Six children reported they had been unable to answer teachers’ questions in class because they were too nervous. However, they now felt more confident about this, thanks to the SEL classes on regulating emotions. A girl aged 12 described this well, “*In the past, if teachers asked me questions in class, I didn’t say anything even if I knew the answer. But now I calm down by taking a deep breath or holding a pen in my hand (like the SEL teacher taught us), and then encourage myself to do it. So I’m able to answer teachers’ questions in a loud voice now.*”

In summary, the SEL classes increased confidence in many children by teaching them to ignore unpleasant comments, to challenge negative thoughts, to develop positive self-talk, and to cope with anxiety. Since the SEL classes, children were more able to act confidently, to say what they think, and to mix with others.

## 4. Discussion

A school-based social-emotional learning (SEL) program was implemented to evaluate its potential to enhance confidence and self-reported self-esteem among children in economically underdeveloped rural regions of China. The key findings from the study are as follows: (1) children from lower socioeconomic backgrounds consistently reported lower levels of self-esteem and self-efficacy across all three assessment points. (2) There was a short-term intervention effect on self-esteem, with greater improvements observed among children from lower-income families. (3) One third of the interviewed children described feeling more confident following the SEL classes. (4) The increase in children’s confidence meant they were more able to mix with others and express themselves, which further improved their relationships with others. (5) According to the teachers, the classroom environment became more lively, and more children answered the teachers’ questions. However, children’s improvements were not consistently sustained up to the 5-month follow-up.

The intervention had a positive effect on self-esteem at the immediate post-intervention. Drawing on the cognitive behavioral approach (Beck & Beck, 2011), the program helped children recognize helpful and unhelpful thoughts and transform unhelpful thoughts into helpful ones, resulting in more positive behaviors (Ahlen et al., 2019). Thus, children who lacked confidence were able to explore their presumptions, weaken negative beliefs, and find positive alternatives, which made them feel good about themselves (Epel et al., 2021). In the SEL classes, role-playing exercises taught children how to express themselves confidently and to “Say no” when they felt uncomfortable. These definitely helped some children develop their confidence and self-esteem (Kouvava et al., 2011). Only one-third of the interviewed children reported that they had benefited from the intervention, describing increases in confidence and self-esteem, while two-thirds of the children experienced no change. Qualitative insights from children further illustrate the mechanisms of change. Those who reported feeling more confident explained that this new sense of self-assurance enabled them to participate more actively in classroom discussions and to build closer peer relationships, suggesting that improved self-esteem facilitated broader social engagement. The reasons underlying why some children benefited from the intervention while others did not are not yet fully understood, but there are clearly highly individual differences in how children respond to such interventions. This study found that although all children came from economically disadvantaged backgrounds, family economic status still moderated the intervention effect, with relatively poorer children (compared to their peers and classmates) showing greater improvements in self-esteem. Prior research has shown that children from higher socioeconomic backgrounds tend to report higher levels of confidence and self-esteem (Twenge & Campbell, 2002). Therefore, children from relatively richer families, who may have already exhibited higher levels of self-esteem before the intervention, might have been less responsive to its effects. Beyond socioeconomic status, several factors may help explain this heterogeneity. Children’s baseline self-esteem levels within the intervention group may have influenced their capacity to benefit, with those starting from lower levels having more opportunity for improvement. Classroom dynamics, including peer relationships and teacher support, may also have shaped the degree to which SEL skills were practiced and reinforced (Wang et al., 2016). Although these factors were not directly assessed in the present study, they represent important avenues for future research to better understand for whom and under what conditions SEL programs are most effective.

Our findings suggest that the increase in children’s confidence and self-esteem enabled them to interact more with others, thus improving their relationships. Within the collectivist Chinese context, children’s confidence improvements often translated into more group participation and peer acceptance, rather than being expressed solely as individual achievements. Consistent with prior research, children with higher self-esteem tend to have more opportunities for socialization, develop stronger friendships, and experience greater social success (Wilkinson & Kao, 2019). Children with high self-esteem believe they have social worth, which leads them to actively build and keep positive social interactions (Marshall et al., 2014). Conversely, individuals with low self-esteem may avoid social connections to protect themselves from rejection, thus failing to establish or maintain social relationships. Self-esteem shapes people’s behavior in social interactions, which has long-term consequences for the quality of their relationships with others (Orth & Robins, 2022).

Three school teachers reported that children were more able to answer questions and communicate with each other, making the classroom environment more lively. Children’s higher self-esteem and confidence in their ability can serve as a motivator and increase their participation in classroom activities (Zhao et al., 2021). However, children’s improvements were not sustained at the 5-month follow-up according to the teachers’ report. There were a number of possible reasons for this. The relative brevity of the program supports previous findings that short-term prevention interventions produce time-limited benefits, whereas long-term programs are more likely to lead to enduring benefits (Clarke et al., 2014). Thus, the positive effects might be sustainable if the SEL classes could continue and be integrated into the school curriculum. In addition, contextual constraints in rural China may have limited the sustainability of the intervention’s effectiveness. In particular, disadvantaged rural schools often face shortages of educational and psychological resources, while the prioritization of academic achievement leaves little room for the practice of SEL skills (Pan & Ye, 2017). Additionally, focusing solely on children without considering family and school factors was insufficient. A more integrated model with a sequenced curriculum implemented throughout the primary school grades, in combination with strategies at the school environment and family level, might lead to more enduring outcomes (Sheffield et al., 2006).

This study shows the appropriateness of the SEL-based program within the context of economically underdeveloped rural China, where academic achievement is prioritized and social-emotional development is often neglected. SEL programs may provide a counterbalance, which helps children better navigate the stress and pressures of the education system, promoting confidence and self-esteem. For example, participants in the intervention school learned coping strategies to deal with their anxiety or nervousness, which enables them to control the situation at hand, and thus feel more confident (Hanton et al., 2004). On the other hand, some caregivers reported they learnt to encourage their children because they knew this was important in the SEL program. According to Bandura’s performance accomplishments mechanism, enhanced efficacy expectations using cognitive strategies such as positive self-talk or external encouragement from significant others protect people against nervousness and anxiety, thereby boosting their confidence and self-esteem (Bandura, 1997). Given this, it is essential for parents and caregivers to be actively involved in creating a supportive environment that promotes children’s academic achievement and self-esteem.

This study has certain limitations. First, the participants were recruited from only two schools, which definitely limits the generalizability of the findings. Second, one facilitator led around 50 children in each classroom, making it challenging to provide adequate attention and support to each individual child. Third, the selection of caregivers may have been biased, as participation was voluntary, meaning those who took part were more likely to be parents of children who had benefited from the intervention. Fourth, each session consisted of two 45 min lessons, conducted one afternoon per week. Some children, particularly those in the lower grades, struggled to maintain focus during the second lesson, potentially compromising the efficacy of the intervention. Finally, the qualitative data provided supportive insights, but retrospective reports may be shaped by recall bias and participants’ expectations (Conway & Ross, 1984).

## 5. Implications for Future Research

More research is needed to improve the effectiveness of SEL programs in improving children’s confidence and self-esteem in economically underdeveloped rural regions of China. Our study has implications for future programs: (1) The two 45 min lessons in each session should be divided and delivered on different days to reduce fatigue and maintain engagement. (2) Programs should be delivered in smaller groups of around 10–12 children, which allows facilitators to provide more individualized support and practice opportunities. Age-tailored adaptations should be considered for future research. (3) Family-inclusive approaches should be strengthened. For example, parent or caregiver sessions could focus on reinforcement strategies such as giving praise and tangible rewards when appropriate (Barrett et al., 2005). Parents or caregivers should also be trained in communication skills, because the positive interactions between caregiver and child are important to help children gain confidence (Laible et al., 2004). (4) Quantitative surveys should incorporate multiple informants, including caregivers and school teachers, to capture more comprehensive perspectives. In addition, follow-up studies are needed to examine whether program effects can be maintained over time. (5) Apart from program facilitators, school teachers should also be trained to ensure that SEL content can be consistently delivered, embedded into daily routines, and sustained over time within the school curriculum.

## 6. Conclusions

These findings highlight the potential value of SEL programs for children in underdeveloped rural areas, where efforts to reduce intergenerational poverty remain challenging. By supporting psychological development, particularly among the most disadvantaged children, such interventions may contribute not only to educational outcomes but also to broader social mobility and poverty reduction. The findings also suggest the need for education policies that integrate SEL into school curricula to promote mental well-being and equitable development. Schools and NGOs could embed SEL into daily classroom routines, provide training for facilitators and school teachers, and involve caregivers to reinforce skills at home, thereby enhancing the sustainability and impact of these programs.

This was essentially a pilot project that demonstrated the feasibility of an SEL-based program in underdeveloped rural China. Further research is needed to enhance the effectiveness of the program before considering its scaling up. The program has potential replicability and sustainability: (1) it is relatively cheap to carry out; (2) it is easily implemented by non-professionals with proper training; (3) it could be conducted in regular school hours, ensuring high attendance, and could be integrated into the curriculum; (4) it is well accepted by children, caregivers and school teachers.

## Figures and Tables

**Table 1:** Baseline sociodemographic and background information by treatment group N (%).

	Total (555)	Control (325)	Intervention (230)	*χ* ^2^	*p*
**Sex**				1.15	0.28
Male	272 (49)	166 (51.1)	106 (46.1)		
Female	283 (51)	159 (48.9)	124 (53.9)		
**Grade**				10.98	0.03
2nd	103 (18.6)	65 (20)	38 (16.5)		
3rd	116 (20.9)	64 (19.7)	52 (22.6)		
4th	143 (25.8)	78 (24)	65 (28.3)		
5th	109 (19.6)	57 (17.5)	52 (22.6)		
6th	84 (15.1)	61 (18.8)	23 (10)		
**Live with**				8.63	0.01
Both parents	326 (58.7)	201 (67.9)	125 (55.8)		
One parent	117 (21.1)	60 (20.3)	57 (25.4)		
Neither parent	77 (13.9)	35 (11.8)	42 (18.8)		
**Father migrant worker**				7.49	0.02
Yes	318 (57.3)	182 (56.7)	136 (59.4)		
No	151 (27.2)	100 (31.2)	51 (22.3)		
Do not know	81 (14.6)	39 (12.1)	42 (18.3)		
**Mother migrant worker**				4.83	0.09
Yes	180 (32.4)	104 (32.5)	76 (33.2)		
No	308 (55.5)	188 (58.8)	120 (52.4)		
Don’t know	61 (11)	28 (8.8)	33 (14.4)		
**Family economic status**				0.002	0.96
Above average	178 (32.1)	104 (32)	74 (32.2)		
Average or below	284 (51.2)	164 (50.5)	120 (52.2)		
**Academic performance**				4.82	0.09
Top 30%	216 (38.9)	130 (40.1)	86 (38.4)		
Middle 50%	211 (38)	114 (35.2)	97 (43.3)		
Bottom 20%	121 (21.8)	80 (24.7)	41 (18.3)		

**Table 2:** Descriptive statistics of self-esteem and self-efficacy.

		**Treatment Group**						
		**Control**		**Intervention**				
		**n**	**Mean (SD)**	**n**	**Mean (SD)**	**t**	**d**	***p***
**Self-esteem**	Assessment 1	320	12.28 (2.61)	229	12.14 (2.8)	0.58	0.05	0.56
	Assessment 2	313	12.63 (2.73)	224	12.96 (2.86)	−1.31	−0.11	0.19
	Assessment 3	325	12.4 (2.93)	230	12 (2.84)	1.58	0.14	0.11
**Self-efficacy**	Assessment 1	322	8.9 (2.27)	225	9.1 (2.34)	−0.75	−0.07	0.46
	Assessment 2	324	9.28 (2.18)	229	9.11 (2.19)	0.87	0.08	0.39
	Assessment 3	325	8.9 (2.35)	230	8.82 (2.15)	0.65	0.06	0.52
		**Family Economic Status**						
		**Above Average**		**Average or Below**				
		**n**	**Mean (SD)**	**n**	**Mean (SD)**	**t**	**d**	***p***
**Self-esteem**	Assessment 1	177	13.08 (2.53)	281	12.04 (2.55)	4.28	0.41	<0.001
	Assessment 2	174	13.36 (2.6)	274	12.59 (2.82)	2.95	0.29	0.003
	Assessment 3	178	12.87 (2.67)	284	12.06 (2.81)	3.13	0.30	0.002
**Self-efficacy**	Assessment 1	177	9.49 (2.27)	279	8.71 (2.28)	3.54	0.34	<0.001
	Assessment 2	178	9.65 (2.19)	283	9.1 (2.12)	2.68	0.26	0.008
	Assessment 3	178	9.39 (2.07)	284	8.79 (2.21)	2.96	0.28	0.003

**Table 3:** Main and interaction effects in the linear mixed models of the intervention on self-esteem and self-efficacy.

Model on Self-Esteem					
**Fixed effect**					
	Coefficient (B)	95%CI	SE	t	*p*
Intervention	−0.16	[−0.63, 0.32]	0.24	−0.65	0.52
Assessment 2	0.24	[−0.08, 0.57]	0.17	1.45	0.15
Assessment 3	0.02	[−0.31, 0.34]	0.16	0.1	0.92
Intervention × Assessment 2	0.57	[0.07, 1.07]	0.25	2.24	0.025
Intervention × Assessment 3	−0.18	[−0.67, 0.31]	0.25	−0.72	0.47
**Random effect**					
		Variance		S.D.	
Participant (Intercept)		3.39		1.84	
Residual		4.1		2.03	
**Model fit**					
R^2^		Marginal		Conditional	
		0.04		0.48	
**Model on self-efficacy**					
**Fixed effect**					
	Coefficient (B)	95%CI	SE	t	*p*
Intervention	0.18	[−0.21, 0.57]	0.2	0.9	0.37
Assessment 2	0.38	[0.08, 0.67]	0.15	2.52	0.01
Assessment 3	0.03	[−0.26, 0.32]	0.15	0.22	0.83
Intervention × Assessment 2	−0.32	[−0.77, 0.13]	0.23	−1.41	0.16
Intervention × Assessment 3	−0.28	[−0.73, 0.17]	0.23	−1.21	0.23
**Random effect**					
		Variance		S.D.	
Participant (Intercept)		1.58		1.26	
Residual		3.4		1.83	
**Model fit**					
R^2^		Marginal		Conditional	
		0.02		0.33	

Note. SE—Standard Error; S.D.—Standard Deviation. Adjusting for sex, grade, and whether living with parents.

## Data Availability

The datasets used during the current study are available from the corresponding author on reasonable request.

## Abbreviations

The following abbreviations are used in this manuscript:

SEL	Social and emotional learning
UNICEF	United Nations International Children’s Emergency Fund
RSES	Rosenberg Self-Esteem Scale
GSES	General self-efficacy scale
LMM	Linear mixed models
